# The role of gratitude in promoting agentic engagement among Chinese EFL learners: a chain mediation study

**DOI:** 10.3389/fpsyg.2026.1830536

**Published:** 2026-06-05

**Authors:** Linyan Hu, Xi Zhang

**Affiliations:** 1 School of Marxism, Jiangxi University of Traditional Chinese Medicine, Nanchang, China; 2 School of Public Policy and Administration, Nanchang University, Nanchang, China

**Keywords:** agentic engagement, Chinese EFL learners, control-value appraisals, enjoyment, gratitude

## Abstract

**Introduction:**

Agentic engagement is fundamental to fostering language proficiency and learner autonomy in language education. Although the positive psychology movement in second language acquisition has increasingly examined how learners' positive traits facilitate general engagement, the specific relationship between gratitude and agentic engagement remains largely unexplored. Grounded in the control-value theory, this study explored how gratitude, control-value appraisals, and foreign language enjoyment collectively predict agentic engagement.

**Methods:**

A total of 3,764 Chinese university students participated in a questionnaire survey. Structural equation modeling was employed to analyze the data.

**Results:**

Results showed that gratitude, control-value appraisals, and enjoyment each positively predicted agentic engagement. Mediation analyses further identified that control-value appraisals and enjoyment mediated the relationships between gratitude and agentic engagement, involving three mediating paths: independent mediation by control-value appraisals, independent mediation by enjoyment, and chain mediation through control-value appraisals and enjoyment.

**Discussion:**

Although the chain mediation effects were modest, these findings illuminate the cognitive and emotional pathways through which gratitude fosters agentic engagement, underscoring the value of integrating gratitude cultivation into EFL instruction. Theoretical and pedagogical implications are discussed.

## 1 Introduction

Learning engagement has attracted substantial research attention in recent years. In the field of second language acquisition (SLA), it has been widely established as a key predictor of learners' academic achievement and language proficiency development (Hiver et al., 2024; Ren, 2023; Zhang and Wang, 2022). The classic model of engagement comprises three dimensions: behavioral, cognitive, and emotional (Fredricks et al., 2004). However, as theoretical perspectives on student agency have advanced, scholars have increasingly critiqued this tripartite model for its implicit portrayal of learners as passive recipients of instruction rather than active contributors to the learning process (Reeve et al., 2020; Reeve and Jang, 2022). In response to this limitation, Reeve (2013) proposed a fourth dimension, agentic engagement, which captures learners' proactive

and constructive contributions to the instructional flow. Unlike the three traditional dimensions, which reflect how students react to teacher-provided activities, agentic engagement refers to students' intentional efforts to personalize and enrich their learning experiences by expressing preferences, offering input, and seeking clarification. This conceptual expansion reflects a broader paradigmatic shift toward recognizing learners as co-constructors of the educational environment (Reeve, 2013). Given that foreign language learning is dependent on learner autonomy and resilience, agentic engagement serves as a crucial catalyst for academic success and sustainable development (Guo and Li, 2018). In contexts where teacher-centered instruction remains prevalent, understanding the antecedents of agentic engagement holds significant pedagogical implications for fostering more self-directed and empowered language learners.

Consequently, SLA researchers have extensively investigated potential antecedents of agentic engagement. Existing research has primarily focused on two categories of influencing factors. The first operates at the situational level, encompassing external supports such as classroom climate, teacher support, and educational technology (Dewaele and Li, 2021; Khajavy et al., 2018; Sadoughi and Hejazi, 2023). The second centers on individual-level factors, including positive personality traits such as grit, growth mindset, and resilience (e.g., Derakhshan et al., 2022; Derakhshan and Noughabi, 2024; Feng and Papi, 2020). Nevertheless, the potential role of gratitude, which is a key positive personality trait, in promoting agentic engagement in language learning has not yet received sufficient scholarly attention. Gratitude is defined as a stable disposition characterized by a tendency to recognize and respond with thankfulness to benevolence, help, and positive experiences received from external sources (Wood et al., 2010). In general education research, empirical evidence consistently points to a significant positive relationship between gratitude and learners' agentic engagement (Jin and Wang, 2019; Zhen et al., 2021). Specifically, learners with a stronger disposition toward gratitude tend to demonstrate higher learning motivation (Cui et al., 2023), more frequent positive emotions (Valdez et al., 2022), and elevated levels of agentic engagement (King et al., 2023; Zhang and Tsai, 2023).

Although the positive relationship between gratitude and agentic engagement has been documented in general educational settings (e.g., Armenta et al., 2022; King et al., 2023), whether this association extends to language learning contexts remains an open question. It is plausible that gratitude also plays a meaningful role in fostering agentic engagement among foreign language learners. At its core, gratitude entails a felt obligation to reciprocate perceived benevolence (Emmons and McCullough, 2003), an impetus that can motivate individuals to invest proactively in the relationships and settings that support them (Bartlett and DeSteno, 2006). Language learning is inherently social, relying heavily on interaction and sustained teacher support (Mercer and Dörnyei, 2020). When learners experience gratitude for such support, the resulting desire to “give back” is likely to manifest as agentic engagement—active, intentional contributions that enrich the learning environment, such as asking questions, proposing ideas, and seeking out learning opportunities (Reeve, 2013). In this way, gratitude may serve as a potent catalyst for the proactive, reciprocal participation that characterizes agentic engagement in the language classroom.

Drawing on control-value theory (Pekrun, 2006), this study conceptualizes gratitude as a distal antecedent that shapes learners' cognitive appraisals of learning activities. Within this framework, positive personality traits such as gratitude are theorized to strengthen perceived control and subjective task value, which in turn function as proximal antecedents of positive achievement emotions—well-established facilitators of agentic engagement in language learning (e.g., Shao et al., 2020; Zhang and Wang, 2023). Guided by this theoretical logic, the study proposes and tests a structural equation model to examine how gratitude influences agentic engagement in English-as-a-foreign-language (EFL) learning among Chinese university students. Specifically, the model evaluates the mediating roles of control-value appraisals and foreign language enjoyment in this pathway. By elucidating these cognitive and emotional mechanisms, this study contributes to a more refined theoretical account of the antecedents of agentic engagement in SLA and offers implications for designing pedagogical interventions that cultivate both proactive learning behaviors and learners' positive psychological traits.

## 2 Literature review

### 2.1 Gratitude and agentic engagement

Agentic engagement is what students say and do to create a more supportive learning environment for themselves (e.g., offer their input, express a preference, and find interesting things to do) (Reeve and Jang, 2022). With the continuous deepening of the “student-focused” approach in foreign language education, agentic engagement has received increasing scholarly attention. Existing research has confirmed that agentic engagement positively influences language learners' willingness to communicate, academic achievement, and wellbeing (e.g., Mercer, 2019; He, 2025; Pan et al., 2023). For instance, Mercer (2019) found that learners who actively engage in their language learning exhibit a stronger willingness to communicate and are better able to overcome language barriers. Drawing on a sample of Chinese EFL learners, He (2025) showed a significant positive correlation between agentic engagement and English achievement. Given its established role in facilitating learning outcomes, recent studies have actively explored the antecedents of agentic engagement from two primary perspectives: the environmental and the individual. At the environmental level, factors such as a positive classroom climate and teacher support have been shown to effectively enhance language learners' agentic engagement (Dewaele and Li, 2021; Sadoughi and Hejazi, 2023). At the individual level, psychological traits such as growth mindset, grit and resilience have also been identified as significant positive predictors of agentic engagement (Derakhshan and Noughabi, 2024; Liu et al., 2024; Yang and Liang, 2025).

Notably, among various positive psychological traits, gratitude has been established in general education research as a key predictor of agentic engagement (e.g., Armenta et al., 2022; Zhen et al., 2021). Within positive psychology, gratitude is commonly conceptualized in two forms: trait gratitude and state gratitude. State gratitude refers to a transient emotional response triggered by a specific positive event, whereas trait gratitude is defined as a more stable, broader life orientation toward noticing and appreciating positivity in daily life (Wood et al., 2010; Măirean et al., 2019). Due to its cross-situational stability and stronger capacity to predict enduring behavioral outcomes, the present study focuses specifically on trait gratitude.

A growing body of research in general education has demonstrated that gratitude can effectively stimulate learners' proactive behaviors and exerts a direct, positive influence on agentic engagement (e.g., Jin and Wang, 2019; Valdez et al., 2022). For instance, Armenta et al. (2022) found that trait gratitude positively predicts students' agentic engagement in academic settings, a finding subsequently replicated by King et al. (2023) across diverse student populations. Collectively, these studies indicate that grateful students are more inclined to actively shape their learning environments and contribute constructively to classroom discourse. Within the field of SLA, however, the potential role of gratitude in fostering agentic engagement has received limited empirical attention. Given the consistent evidence emerging from general education, coupled with the inherently interactive and socially embedded nature of language classrooms (MacIntyre et al., 2016), it is plausible that gratitude functions as an especially potent catalyst for agentic engagement in EFL contexts. Extending this line of reasoning, we propose the following hypothesis:

H1. Gratitude would predict EFL learners' agentic engagement positively.

### 2.2 Control-value appraisals as mediators

Control appraisal refers to a learner's belief and judgment about their ability to successfully master or complete a given learning task, whereas value appraisal pertains to their perception of the task's importance, utility, or intrinsic meaning (Pekrun et al., 2007). Value appraisal can be further distinguished into intrinsic value (stemming from interest in, enjoyment of, or personal identification with the learning activity itself) and extrinsic value (related to the pragmatic benefits or achievement-related outcomes associated with the task) (Pekrun et al., 2007).

Recent SLA research has consistently demonstrated that both control and value appraisals directly shape learning engagement (Shao et al., 2019; Beymer et al., 2023; Xu and Qiu, 2025). For instance, Shao et al. (2020) found that EFL learners who felt confident in their abilities and recognized the value of language learning tended to adopt deep learning strategies and sustain their engagement. Studies focusing specifically on agentic engagement have reinforced this pattern, confirming that control and value appraisals serve as direct positive predictors of agentic engagement among foreign language learners (Bordbar, 2021; Xu et al., 2023; Zhao et al., 2024).

Moreover, evidence from general educational psychology further suggests that such cognitive appraisals can be cultivated by dispositional gratitude (Liao, 2016; Li, 2021). Students with a stronger grateful disposition are more likely to view their learning capabilities in a positive light, thereby exhibiting a heightened sense of academic control (Tang and Ge, 2014). Concurrently, the social–cognitive dimension of gratitude—a sense of moral obligation or a desire for reciprocity—can reframe learning as an act of reciprocation or honor toward benefactors (e.g., parents and teachers). This reframing imbues learning tasks with greater personal and social meaning, strengthening both intrinsic and extrinsic valuations of the activity (Liao, 2016; Naito et al., 2005). Taken together with the established link between appraisals and agentic engagement, these findings suggest a plausible mediating role of control-value appraisals in the relationship between gratitude and agentic engagement. Therefore, we propose the following hypotheses:

H2. Control appraisals would mediate the relationship between gratitude and agentic engagement.
H3. Value appraisals would mediate the relationship between gratitude and agentic engagement.

### 2.3 Foreign language enjoyment as mediators

According to broaden-and-build theory (Fredrickson, 2004), positive emotions broaden individuals' momentary thought–action repertoires and build enduring personal resources. In SLA, this framework has been widely applied to explain how positive academic emotions facilitate learning, with evidence indicating that they enhance attention, cognitive flexibility, and agentic engagement (Dewaele and MacIntyre, 2014; Jiang and Dewaele, 2019; Shao et al., 2020). Foreign language enjoyment—defined as the pleasure, joy, and satisfaction derived from language learning, use, or communication (Dewaele and MacIntyre, 2014)—is a prototypical positive emotion in this context. A substantial body of SLA research has consistently shown that enjoyment not only mitigates anxiety and optimizes cognitive resource allocation, but also strengthens intrinsic motivation and agentic engagement (Jiang and Dewaele, 2019; Zhang and Wang, 2023). Consistent with broaden-and-build theory, enjoyment thus supplies the intrinsic drive for sustained, agentic engagement (Diener et al., 2020; Fredrickson and Joiner, 2018).

Previous research has also identified gratitude as a significant antecedent of positive emotions, particularly enjoyment (Dickens, 2017; Yoo, 2020). Individuals higher in dispositional gratitude typically report stronger enjoyment and greater subjective wellbeing (Yoo, 2020), and longitudinal evidence shows that gratitude directly and stably predicts enjoyment (Liang et al., 2015). Within the SLA context, studies demonstrate that gratitude is negatively associated with anxiety and positively associated with enjoyment (Jin et al., 2021; Scida and Jones, 2017). Collectively, these findings indicate that learners with a stronger sense of gratitude are more prone to experience and sustain enjoyable emotional states during language learning. Integrating broaden-and-build theory with this evidence, we propose the following hypothesis:

H4. Foreign language enjoyment would mediate the relationship between gratitude and agentic engagement.

### 2.4 Control-value appraisals and enjoyment as chain mediators

According to control-value theory, individuals' appraisals of control over and value of achievement activities serve as proximal antecedents of academic emotions, and they can be leveraged to understand and promote affective growth (Pekrun, 2006, 2023). In language learning contexts, learners who perceive greater control and higher value in their foreign language study tend to experience heightened foreign language enjoyment (Pekrun, 2006; Shao et al., 2020). This pathway has received robust empirical support in SLA. For example, Zou et al. (2025) showed that control-value appraisals significantly predicted Chinese EFL learners' agentic engagement, primarily through the mediating role of enjoyment, and Xu and Qiu (2025) found that positive high-arousal emotions—especially enjoyment—served as a significant indirect link between appraisals and EFL reading engagement.

Despite these advances, trait gratitude has not yet been integrated with control-value appraisals, enjoyment, and agentic engagement within a unified chain mediation framework in SLA. Prior research has examined these variables largely in isolation or in narrower configurations, leaving their sequential interplay largely unaddressed. Synthesizing the evidence reviewed above, a coherent theoretical account can be tentatively articulated: gratitude may function as a distal catalyst that enhances perceived control over academic outcomes and increases the subjective valuation of learning. Strengthened appraisals, in turn, predispose learners to more frequent and intense episodes of foreign language enjoyment, consistent with control–value theory (Pekrun, 2006). This heightened enjoyment, together with the motivational impetus derived directly from the appraisals themselves (Shao et al., 2019; Xu et al., 2023), can foster greater agentic engagement. Critically, this line of reasoning delineates two distinct yet parallel cognitive-emotional pathways through which gratitude may promote agentic engagement. Accordingly, we advance the following chain mediation hypotheses:

H5. Control appraisals and enjoyment would mediate the relationship between gratitude and agentic engagement.
H6. Value appraisals and enjoyment would mediate the relationship between gratitude and agentic engagement.

## 3 The study

### 3.1 Conceptual model

**Figure 1:**
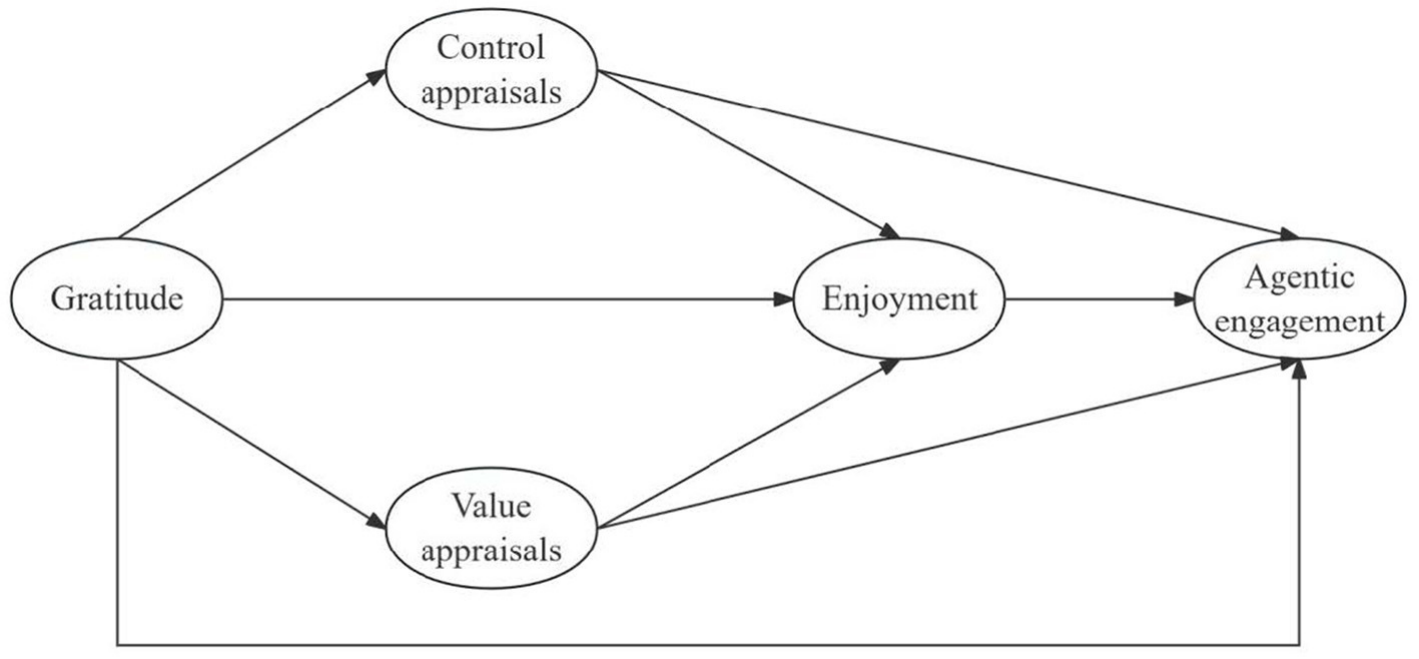
Conceptual model.

Informed by the identified gaps in the existing literature, this study proposes a conceptual model to examine the mechanism through which gratitude impacts agentic engagement in EFL learning context. As depicted in Figure 1, the model is based on an integration of the control-value theory and the broaden-and-build theory. It posits that learners' agentic engagement is not only directly influenced by their dispositional gratitude but also indirectly affected through a series of cognitive and affective pathways. The primary objective of this model is to untangle the specific processes connecting gratitude to agentic engagement. It explores the mediating role of control-value appraisals and the subsequent mediating role of foreign language enjoyment, thus testing a potential chain of influence. This approach endeavors to clarify how a grateful disposition may first enhance learners' perceptions of competence and task value, which in turn could promote positive emotions, ultimately culminating in greater agentic engagement in language learning.

### 3.2 Participants

A pilot study was initially conducted to refine the questionnaire, during which ambiguous or misleading items were revised. An attention-check question was also embedded to facilitate the filtering of invalid responses. For the formal survey, a convenience sampling method was adopted. Participants were non-English-major undergraduate students recruited from 12 universities spanning northern, eastern, central, southern, and southwestern China. Data were collected via Wenjuanxing, a professional online survey platform widely used in China. At each university, four intact classes were randomly selected. A total of 4,050 electronic questionnaires were distributed. After removing invalid responses (e.g., incomplete submissions and patterned answering), 3,764 valid questionnaires were retained, yielding an effective response rate of 92.94%.

The final sample comprised 1,827 male (48.54%) and 1,937 female (51.46%) students, with a mean age of 20.5 years (SD = 2.31). Regarding place of origin, 2,242 (59.56%) participants were from urban areas, while 1,522 (40.44%) were from rural areas. A majority (81.80%) had begun learning English in primary school, resulting in an average of over 9 years of English instruction. By academic discipline, 44.18% of participants were enrolled in Natural Sciences and 55.82% in Humanities and Social Sciences. All participants were informed of the study's purpose and provided voluntary consent. The questionnaire was administered in Chinese, their native language, to ensure clarity and accurate comprehension.

### 3.3 Measurement instruments

All instruments used in this study were originally developed in English and underwent a rigorous translation and back-translation procedure to ensure semantic and conceptual equivalence (Brislin, 1980). Drawing on a comprehensive literature review and pilot analyses, we constructed a structured questionnaire comprising two sections. The first section collected participants' demographic information (e.g., gender, university year, place of origin, and major), and the second assessed the focal variables under investigation—namely, gratitude, control appraisal, value appraisal, foreign language enjoyment, and agentic engagement (see Supplementary File S1 for the full list of items). All items were rated on a five-point Likert scale ranging from 1 (strongly disagree) to 5 (strongly agree).

#### 3.3.1 Gratitude scale

To better account for enduring individual differences in agentic engagement, trait gratitude was selected as the core antecedent variable, as it reflects a relatively stable psychological tendency (Wood et al., 2010). The measurement was adapted from the Gratitude Questionnaire-6 (GQ-6; Emmons and McCullough, 2003), a six-item scale assessing trait gratitude. The GQ-6 has demonstrated good reliability and validity among Chinese student populations (Yang et al., 2021; Cui et al., 2023; Zhang et al., 2021). Items were contextualized for English language learners (e.g., “I am grateful to those who have supported me in my English learning”). In the present study, the scale showed excellent internal consistency, with a Cronbach's α of 0.95. The results of the confirmatory factor analysis (CFA) were satisfactory (χ^2^/df = 7.07, CFI = 0.99, TLI = 0.99, RMSEA = 0.04, and SRMR = 0.02).

#### 3.3.2 Measures of control-value appraisals

Control appraisal was measured using a scale adapted from Perry et al.'s (2001) Academic Control Scale. The adapted version consists of three items (e.g., “The harder I work in English class, the better my grades will be”), translated and contextualized for the EFL learning setting. Reliability analysis indicated good internal consistency, with a Cronbach's α of 0.93. The CFA produced satisfactory results (χ^2^/df = 5.78, CFI = 0.99, TLI = 0.99, RMSEA = 0.04, and SRMR = 0.02). The value appraisal scale was adapted from the task value subscale of the motivated strategies for learning questionnaire (MSLQ) (Pintrich et al., 1993). The adapted six-item scale assesses two dimensions: intrinsic value (e.g., “I find the content learned in English class interesting”) and extrinsic value (e.g., “Getting a good grade is my main concern in English class”). The full scale and the two subscales all demonstrated good reliability, with Cronbach's α of 0.92, 0.85, and 0.86, respectively. CFA supported the scale's structural validity, with satisfactory model fit indices (χ^2^/df = 5.85, CFI = 0.99, TLI = 0.99, RMSEA = 0.04, and SRMR = 0.01).

#### 3.3.3 Foreign language enjoyment scale

Foreign language enjoyment was measured using the Chinese version of the Foreign Language Enjoyment Scale (CFLES; Li et al., 2018), which has been widely validated among Chinese EFL learners (e.g., Lin and Wang, 2025; Zou et al., 2025). The 11-item scale comprises three dimensions: personal enjoyment (e.g., “I enjoy learning English”), teacher appreciation (e.g., “My English teacher always encourages us”), and environmental enjoyment (e.g., “There is a good atmosphere for learning English around me”). In this study, the full scale and subscales showed good to excellent reliability, with Cronbach's α of 0.95, 0.92, 0.87, and 0.79, respectively. CFA results confirmed the scale's structural validity, with acceptable model fit (χ^2^/df = 4.27, CFI = 0.99, TLI = 0.99, RMSEA = 0.03, and SRMR = 0.01).

#### 3.3.4 Agentic engagement scale

Agentic engagement was evaluated using a scale developed by Reeve and Tseng (2011), which is composed of five items. This scale has been extensively utilized to measure agentic engagement among language learners and has proven to have good reliability (e.g., Jiang and Zhang, 2021; Bordbar, 2021). The items were adjusted for the EFL context (e.g., “In English class, I proactively ask questions”). In the current sample, the scale exhibited good internal consistency, with a Cronbach's α of 0.76. The CFA demonstrated good model fit (χ^2^/df = 6.50, CFI = 0.99, TLI = 0.99, RMSEA = 0.04, and SRMR = 0.01).

We further assessed the instruments' convergent and discriminant validity. Convergent validity was evaluated using composite reliability (CR) and average variance extracted (AVE). As Table 1 shows, CR values ranged from 0.80 to 0.95 (above the 0.70 threshold) and AVE values from 0.50 to 0.84, indicating satisfactory convergent validity. Discriminant validity was examined with the Fornell–Larcker criterion: for each construct, the square root of its AVE (diagonal) was greater than its correlations with all other constructs (off-diagonal), confirming adequate distinctiveness among gratitude, control appraisal, value appraisal, foreign language enjoyment, and agentic engagement.

**Table 1:** Summary of convergent and discriminant validity.

Variable	CR	AVE	1	2	3	4	5
Gratitude	0.95	0.75	**0.87**				
Control appraisal	0.93	0.82	0.60	**0.91**			
Value appraisal	0.91	0.84	0.46	0.48	**0.92**		
Enjoyment	0.92	0.79	0.60	0.50	0.41	**0.89**	
Agentic engagement	0.80	0.50	0.64	0.63	0.65	0.55	**0.71**

Values on the diagonal (in bold) are the square root of the AVE for each construct; off-diagonal values are inter-construct correlations.

### 3.4 Data analysis

Data were analyzed using SPSS 26.0 and AMOS 23.0 in three steps. First, to rule out potential inflation from self-report data, common method bias was assessed via the unmeasured latent method construct (ULMC) technique prior to hypothesis testing. Second, preliminary analyses were conducted, including normality tests, descriptive statistics, independent-samples *t*-tests and one-way ANOVAs to identify demographic covariates, and Pearson correlations among the key variables (gratitude, control-value appraisals, enjoyment, and agentic engagement). Third, the hypothesized model was tested with maximum likelihood estimation in AMOS 23.0 to examine direct paths (H1). Bootstrapping procedures (5,000 resamples) were then employed, and bias-corrected 95% confidence intervals were computed to assess simple mediation (H2–H4) and chain mediation (H5–H6) effects, thereby determining whether control-value appraisals and enjoyment serially transmitted the effect of gratitude on agentic engagement.

The model fit was tested using the indices recommended by Kline (2015). Specifically, a CMIN/DF value ≤ 5 indicates a good fit, and values ≤ 8 are acceptable. For RMSEA, values ≤ 0.05 are considered good, while values ≤ 0.08 are acceptable. SRMR values ≤ 0.05 indicate a good fit, and values ≤ 0.08 are acceptable. CFI and TLI values between 0.90 and 0.95 are acceptable, and values above 0.95 indicate a good-fitting model.

### 3.5 Common method bias assessment

To address potential common method bias, an ULMC approach was employed. A six-factor model incorporating a common method factor was compared with the original five-factor measurement model (comprising gratitude, control appraisal, value appraisal, foreign language enjoyment, and agentic engagement). The results indicated that the inclusion of the common method factor did not yield a significant improvement in model fit (ΔCFI = 0.01, ΔTLI = 0.01, and ΔRMSEA = 0.01), suggesting that common method variance does not pose a substantial threat to the validity of the findings in this study (Chen, 2007; Tang and Wen, 2020).

## 4 Results

### 4.1 Descriptive and correlation analyses

Table 2 presents the results of the normality assessment and descriptive statistics. The skewness and kurtosis values indicated that the data were normally distributed (Skewness: between −2 and +2; Kurtosis: between −7 and +7) (West et al., 1995). As shown in Table 2, participants reported a moderate to high level of agentic engagement in EFL learning (*M* = 3.72, SD = 0.59). To further explore potential group differences in agentic engagement, independent-samples *t*-tests and one-way ANOVAs were conducted across various demographic variables. A significant difference was found based on participants' place of origin. Specifically, learners from urban areas demonstrated significantly higher levels of agentic engagement than their counterparts from rural areas (*t* = 8.35, *p* < 0.01). However, no statistically significant differences in agentic engagement were observed in relation to gender, years of English learning experience, academic major (all *ps* > 0.05).

**Table 2:** Descriptive analyses of all the variables (*N* = 3,764).

Variable	Mean	SD	Skewness (SE)	Kurtosis (SE)
Gratitude	3.53	1.00	−1.05 (0.04)	0.24 (0.08)
Control appraisal	3.69	1.09	−1.06 (0.04)	0.42 (0.08)
Value appraisal	3.69	0.84	−0.88 (0.04)	0.39 (0.08)
Enjoyment	3.68	0.79	−1.04 (0.04)	0.61 (0.08)
Agentic engagement	3.72	0.59	−0.44 (0.04)	3.01 (0.08)

As shown in Table 3, all variables were significantly intercorrelated. Gratitude correlated strongly with control appraisal (*r* = 0.60, *p* < 0.01), value appraisal (*r* = 0.46, *p* < 0.01), foreign language enjoyment (*r* = 0.60, *p* < 0.01), and especially agentic engagement (*r* = 0.64, *p* < 0.01). Control and value appraisals also correlated moderately to strongly with enjoyment and agentic engagement. Enjoyment further correlated significantly with agentic engagement (*r* = 0.55, *p* < 0.01), suggesting that positive emotions foster more proactive learning behaviors.

**Table 3:** Correlation coefficients among all the variables.

Variable	1	2	3	4	5
Gratitude	–				
Control appraisal	0.60^**^	–			
Value appraisal	0.46^**^	0.48^**^	–		
Enjoyment	0.60^**^	0.50^**^	0.41^**^	–	
Agentic engagement	0.64^**^	0.63^**^	0.65^**^	0.55^**^	–

^**^p < 0.01.

### 4.2 Direct effects of gratitude on agentic engagement

Independent-samples *t*-tests indicated that urban learners reported significantly higher agentic engagement than their rural counterparts (*t* = 8.35, *p* < 0.01), in line with broader evidence that urban–rural background can shape academic engagement (Pike et al., 2012; Wang et al., 2024). To evaluate potential confounding, we first estimated a structural model that included place of origin (urban = 1, rural = 0) as an exogenous predictor of agentic engagement. The path from place of origin to agentic engagement was small but significant (β = 0.07, 95% CI [0.05, 0.09]). The initial theoretical model exhibited a marginally acceptable yet improvable fit (χ^2^/df = 6.96, RMSEA = 0.04, CFI = 0.98, TLI = 0.98, and SRMR = 0.12). Inspection of modification indices revealed a substantial residual covariance between two indicators (e20 and e21, M.I. = 271.26). After freely estimating this error covariance, the final model demonstrated good fit across all indices (χ^2^/df = 5.05, RMSEA = 0.03, CFI = 0.99, TLI = 0.99, and SRMR = 0.05), meeting established thresholds (Kline, 2015). This final model accounted for 61.80% of the variance in agentic engagement, indicating strong explanatory power.

**Figure 2:**
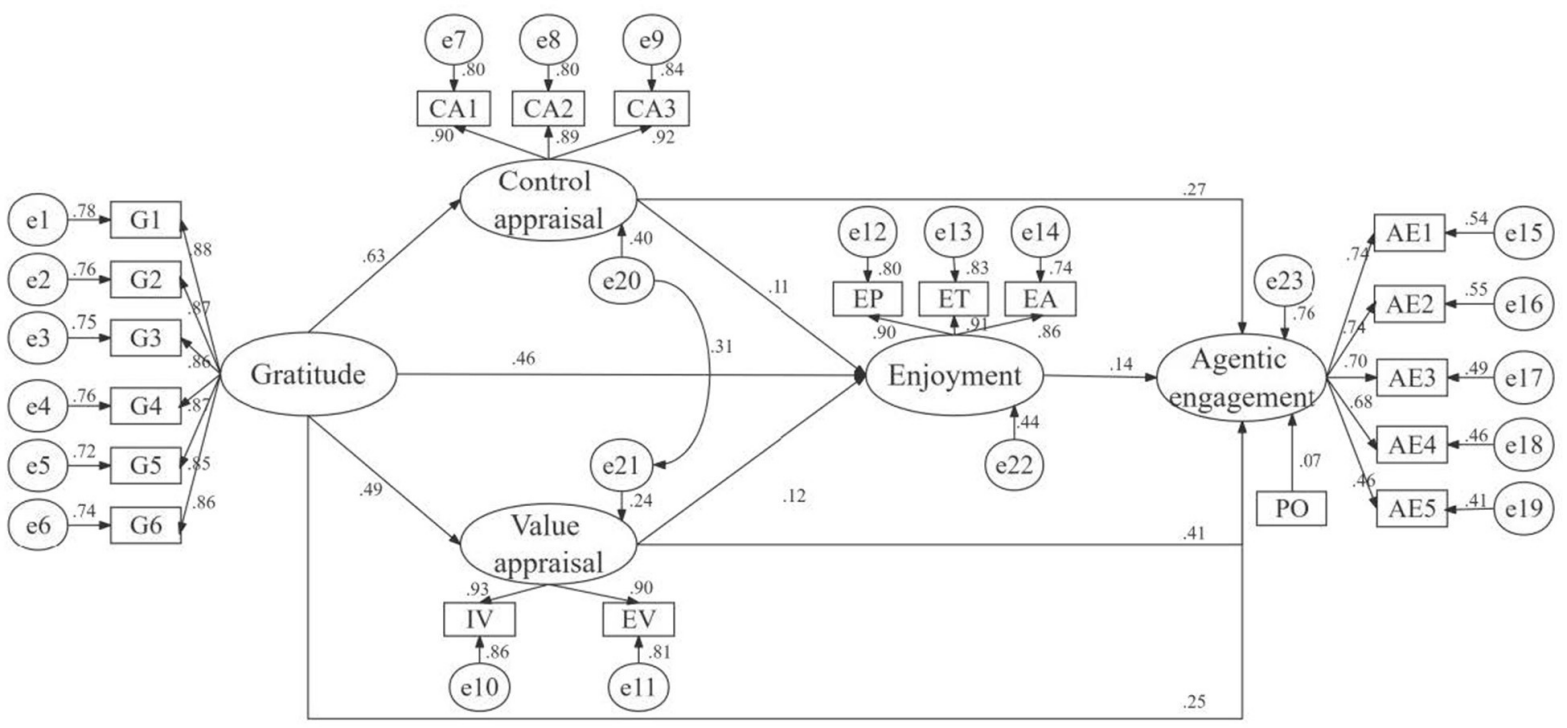
Path diagram of structural equation model. G, gratitude; CA, control appraisal; IV, intrinsic value; EV, extrinsic value; EP, enjoyment-private; ET, enjoyment-teacher; EA, enjoyment-atmosphere. AE, agentic engagement; PO, place of origin. All coefficients were significant with *p*s < 0.01.

As shown in Figure 2, gratitude had a significant positive direct effect on agentic engagement (β = 0.25, 95% CI [0.22, 0.28]), supporting H1. Both control appraisal (β = 0.27, 95% CI [0.25, 0.30]) and value appraisal (β = 0.41, 95% CI [0.38, 0.43]) positively predicted agentic engagement, and foreign language enjoyment also had a significant positive direct effect (β = 0.14, 95% CI [0.11, 0.17]). These significant paths are consistent with the control-value theory premise that cognitive appraisals and achievement emotions act as proximal determinants of engagement (Pekrun, 2006; Shao et al., 2020).

### 4.3 Mediating effects of control-value appraisals and enjoyment between gratitude and agentic engagement

Bias-corrected bootstrapping with 5,000 resamples was further employed to examine the indirect effects of gratitude on agentic engagement via control-value appraisals and enjoyment. As shown in Table 4, all hypothesized indirect pathways were statistically significant, with the 95% bias-corrected confidence intervals excluding zero. Specifically, the indirect effect of gratitude through control appraisals accounted for 24.15% of the total effect (β = 0.17, 95% CI [0.154, 0.188]), supporting H2. The indirect pathway via value appraisals represented 28.53% of the total effect (β = 0.20, 95% CI [0.185, 0.217]), confirming H3. Moreover, foreign language enjoyment independently mediated the relationship, with this indirect effect constituting 8.90% of the total effect (β = 0.06, 95% CI [0.051, 0.077]), providing evidence for H4.

**Table 4:** Summary of the mediation analysis.

Paths	SE	β	*p*	95% CI
Gratitude → control appraisal → agentic engagement	0.01	0.17	0.01	[0.154, 0.188]
Gratitude → value appraisal → agentic engagement	0.01	0.20	0.01	[0.185, 0.217]
Gratitude → enjoyment → agentic engagement	0.01	0.06	0.01	[0.051, 0.077]
Gratitude → control appraisal → enjoyment → agentic engagement	0.00	0.02	0.01	[0.011, 0.020]
Gratitude → value appraisal → enjoyment → agentic engagement	0.00	0.01	0.01	[0.006, 0.011]

Turning to the chain mediation pathways, the sequential indirect effect from gratitude to agentic engagement through control appraisals and enjoyment was significant (β = 0.02, 95% CI [0.011, 0.020]), accounting for 2.26% of the total effect and supporting H5. This finding aligns with the cognitive-emotional sequence posited by control-value theory (Pekrun, 2006). Similarly, the chain mediation via value appraisals and enjoyment was also significant (β = 0.01, 95% CI [0.006, 0.011]), explaining 1.13% of the total effect and further corroborating H6. Taken together, the data support all five hypothesized indirect pathways.

## 5 Discussion

Drawing on control-value theory, the present study explored the relationship between gratitude on agentic engagement among Chinese EFL learners, revealing the cognitive and emotional mechanisms through which gratitude influenced agentic engagement via control-value appraisals and enjoyment. The results are explained as follows.

### 5.1 Direct predictors of agentic engagement

A notable finding is the significant and positive direct effect of gratitude on EFL learners' agentic engagement (β = 0.25, *p* < 0.01), supporting H1. This aligns with prior work positioning gratitude as a facilitator of agentic engagement (Zhao et al., 2024) and extends the association to foreign language learning. Within the Chinese cultural milieu, gratitude embodies a moral orientation rooted in the Confucian norm of “repaying kindness.” Learners with heightened dispositional gratitude tend to internalize this orientation as academic accountability and sustained motivation (Fu and Zhang, 2022; Ye et al., 2023). More concretely, gratitude appears to help learners translate perceived social support into self-directed behaviors—active classroom participation, pursuit of challenging tasks, and flexible strategy regulation—hallmarks of elevated agentic engagement (Guo et al., 2023; Ye et al., 2013; Yost-Dubrow and Dunham, 2018).

Both control appraisal (β = 0.27, *p* < 0.01) and value appraisal (β = 0.41, *p* < 0.01) also significantly predicted agentic engagement. These findings are consistent with control-value theory, which identifies perceived control and subjective task value as proximal determinants of engagement (Pekrun, 2006). Among the Chinese university EFL learners surveyed, those who expressed confidence in their capacity to master English tasks (high control) and who regarded those tasks as personally meaningful or instrumental for future pursuits (high value) were more disposed to demonstrate agentic behaviors. Instead of passively fulfilling assigned requirements, they were more likely to raise questions, articulate preferences, and embrace intellectual challenges (Xu and Qiu, 2025; Shao et al., 2020). Beyond theoretical corroboration, these results suggest that educators may strengthen agentic engagement by deliberately cultivating control beliefs and task value perceptions.

Foreign language enjoyment further exhibited a significant positive direct effect on agentic engagement (β = 0.14, *p* < 0.01). This finding resonates with evidence that positive emotions fuel agentic involvement (Feng and Hong, 2022; Dewaele et al., 2023; Lin and Wang, 2025) and aligns with the broaden-and-build framework (Fredrickson, 2004). According to this perspective, enjoyment broadens thought-action repertoires and builds enduring resources, fostering proactive participation. In language classrooms characterized by trust and emotional support, such positive affect can motivate learners to invest effort and take initiative (Liang and Reiss, 2025). These observations underscore that positive emotions actively foster deeper, more agentic engagement and highlight the value of cultivating affective experiences to enhance learner agency.

### 5.2 Mediating roles of control-value appraisals and enjoyment

The mediation analysis confirmed that the relationship between gratitude and agentic engagement was mediated through control-value appraisals and foreign language enjoyment. In support of H2 and H3, both control appraisal (β = 0.17, *p* < 0.01) and value appraisal (β = 0.20, *p* < 0.01) were found to function as significant mediators. These findings illuminate the sequential cognitive-emotional pathways through which trait gratitude translates into proactive learning behaviors. Importantly, they demonstrate that gratitude not only fosters agentic engagement in isolation; rather, it operates via the synergistic interplay of cognitive reappraisal and emotional experience, thereby extending control-value theory (Pekrun, 2006) to the dispositional antecedents of agentic engagement in SLA.

Central to this process are the control-value appraisals, which serve as the cognitive linchpin of the mediation chain. Gratitude appears to enhance these appraisals through dual mechanisms. On the one hand, grateful learners tend to attribute their academic progress to the intentional support of others (e.g., teachers' encouragement, peers' assistance). This relational attribution reinforces learners' sense of competence and academic control by making them aware of the reliable social resources they can draw upon when facing challenges, a mechanism consistent with the positive attributional style and enhanced coping efficacy linked to gratitude (Liao, 2016; Ye et al., 2013). On the other hand, the inherently relational nature of gratitude may lead learners to reframe learning as a meaningful activity imbued with social significance; studying diligently becomes a way of reciprocating the care and investment received from others, thereby amplifying both intrinsic and extrinsic task value (Li, 2021; Naito et al., 2005). In turn, these heightened value appraisals are likely to fuel greater agentic engagement, as learners proactively invest cognitive and behavioral resources in activities they perceive as personally and socially meaningful (Zhang and Wang, 2023). Notably, the indirect effect through value appraisal was slightly larger than that through control appraisal, suggesting that, within foreign language learning contexts, learners' subjective valuation of tasks may act as a particularly potent driver of agentic engagement. This asymmetry likely reflects China's examination-oriented EFL context, where English serves as a high-stakes gatekeeper to academic and career advancement; consequently, its perceived instrumental and intrinsic value may exert a stronger motivational pull toward agentic engagement than perceived control does (Xu and Qiu, 2025). This finding underscores the practical importance of cultivating value-laden perceptions—for instance, by linking language learning to personal growth and social connectedness. This finding underscores the practical importance of cultivating value-laden perceptions—for instance, by linking language learning to personal growth and social connectedness.

The mediating role of foreign language enjoyment in the gratitude-agentic engagement link was statistically significant (β = 0.06, *p* < 0.01), supporting H4; however, the small effect size should be interpreted with caution. Learners with a stronger grateful disposition are thus only somewhat more likely to experience positive emotions during EFL learning that translate into greater agentic engagement. Enjoyment, therefore, appears to represent one of several affective mechanisms rather than a primary channel. From a broaden-and-build perspective (Fredrickson, 2004), gratitude-elicited enjoyment may serve as a subtle emotional buffer that partially alleviates exhaustion and supports proactive classroom participation, particularly within the anxiety-prone and uncertain context of foreign language learning (Dewaele and MacIntyre, 2014). Although modest, this indirect pathway suggests that gratitude can gently foster the positive emotions conducive to self-initiated learning. This finding aligns with evidence on the affective benefits of gratitude in education (Lin, 2019; Valdez et al., 2022), while extending these benefits to agentic engagement in SLA. Moreover, it resonates with recent SLA studies identifying enjoyment as a mediator between learner dispositions and engagement (Zou et al., 2025; Xu and Qiu, 2025); the small effect size further implies that additional cognitive and motivational mechanisms likely operate in parallel to shape agentic engagement.

Moreover, this study identified significant chain mediation effects, whereby gratitude was sequentially linked to agentic engagement via control-value appraisals and foreign language enjoyment (β = 0.02, *p* < 0.01; β = 0.01, *p* < 0.01), supporting H5 and H6. These indirect effects were statistically significant but small in magnitude, indicating a modest sequential pathway. A plausible explanation for the limited effects is that, according to control-value theory, cognitive appraisals are not merely antecedents of emotions; they also directly influence engagement and performance (Pekrun, 2006; Shao et al., 2019; Xu et al., 2023). Grateful learners may derive considerable motivational impetus directly from heightened perceptions of control and value, without this influence needing to be fully channeled through enjoyment. Consequently, the sequential pathway through control-value appraisals to enjoyment accounts for only a modest incremental portion of the total effect.

Notably, the direct effect of gratitude (β = 0.25) exceeded that of any single indirect path, underscoring its substantial direct motivational role alongside cognitive and emotional pathways. Consistent with prior research (e.g., Cui et al., 2023; Zhen et al., 2021), this finding suggests that gratitude may foster a focused, persistent disposition that translates directly into agentic engagement. The strength of this direct pathway highlights the value of cultivating gratitude as a relatively immediate resource for promoting learner agency in EFL contexts.

## 6 Implications and limitations

This study examined the direct and indirect pathways linking gratitude, control-value appraisals, and foreign language enjoyment to agentic engagement, elucidating the cognitive and motivational mechanisms involved. While prior general education research has established a positive link between gratitude and agentic engagement (e.g., King et al., 2023; Valdez et al., 2022), this relationship has remained largely unexamined in SLA. Our findings position gratitude as a meaningful dispositional trait, highlighting its role as a personality-based resource with sustained downstream effects on agentic engagement, rather than merely a transient emotion. Moreover, by integrating control-value theory (Pekrun, 2006) and broaden-and-build theory (Fredrickson, 2004) into a unified sequential framework, this study provides a coherent theoretical account of how a moral-affective trait translates into agentic forms of classroom participation. Although the chain mediation effects were small, their statistical significance points to a plausible cognitive-emotional mechanism: trait gratitude shapes how learners appraise their control over and the value of language learning, which in turn evokes enjoyment that broadens thought-action repertoires and fosters agentic engagement. These findings thus offer a more nuanced explanatory lens than prior models that examined these variables in isolation.

Our findings also provide pedagogical implications for language educators. First, considering that gratitude interventions can improve learning outcomes partly by enhancing felt social support, academic motivation, and the desire to give back (Valdez et al., 2022), teachers might consider embedding brief gratitude exercises into classroom routines. For instance, following the structured intervention in Valdez et al. (2022), teachers could ask students to spend 5 min at the end of each week writing a short thank-you note to someone who has supported their English learning—a peer, teacher, or family member—thereby cultivating gratitude through repeated reflective acts. Second, to support learners' control appraisals, instructors may wish to provide growth-oriented feedback that emphasizes effort and strategy use, and to design scaffolded tasks with appropriately graded difficulty. Prior work suggests that EFL learners who perceive greater control tend to adopt deeper learning strategies and sustain higher engagement (Shao et al., 2020). Third, given the direct and indirect effects of control-value appraisals on agentic engagement observed in this study, incorporating interventions that target control-value appraisals into the EFL curriculum appears warranted. In particular, instructors could seek to enhance value appraisals by connecting English content to learners' personal interests, career aspirations, and authentic language use, making language learning more personally meaningful and instrumentally valuable (Zhang et al., 2025).

Our study shows its limitations. First, the cross-sectional design constrains causal inferences. Although the mediating pathways are theoretically grounded, the temporal ordering among gratitude, appraisals, enjoyment, and agentic engagement could not be empirically established; the proposed directionality therefore remains tentative. Second, data were collected exclusively through self-report questionnaires. Despite statistical tests indicating no severe common method bias, self-reports are inherently susceptible to social desirability and recall inaccuracies. Future research could adopt longitudinal designs and incorporate multiple data sources-such as classroom observations or digital trace data-to strengthen causal evidence and enhance measurement validity. Third, as the sample comprised exclusively non-English-major undergraduates, the findings may not extend to English majors, whose motivational profiles and instructional contexts typically differ. This boundary condition awaits direct testing through replication with English-major cohorts.

## 7 Conclusion

Drawing on control-value theory (Pekrun, 2006) and broaden-and-build theory (Fredrickson, 2004), this study used structural equation modeling to analyze questionnaire data from 3,764 Chinese university EFL learners, examining how gratitude predicts agentic engagement. Results showed a significant direct positive effect of gratitude on agentic engagement, confirming gratitude as a key dispositional resource that fuels learners' proactive participation. Gratitude also influenced agentic engagement indirectly: control appraisals, value appraisals, and enjoyment each served as significant independent mediators, and two chain mediation pathways—one via control appraisals and enjoyment, the other via value appraisals and enjoyment—were also supported. Importantly, gratitude influenced agentic engagement predominantly through its direct effect and the independent cognitive pathways via control-value appraisals, while the emotional pathway through enjoyment and the chain pathways played auxiliary roles. This study elucidates the mechanisms through which gratitude fosters agentic engagement, contributing to the limited research in the Chinese EFL context. The findings also extend SLA research on the antecedents of agentic engagement and offer new empirical insights for integrating positive psychology into language education.

## Data Availability

The raw data supporting the conclusions of this article will be made available by the authors, without undue reservation.
