# Supplementary material for: The role of gratitude in promoting agentic engagement among Chinese EFL learners: a chain mediation study
Source: Front Psychol. 2026 Jun 5;17:1830536. doi: 10.3389/fpsyg.2026.1830536 (PMC13279615; doi:10.3389/fpsyg.2026.1830536)
Supplement: Supplementary file 1 [file Supplementary_File_1.docx]

**Appendix: Questionnaire on agentic engagement and its influencing factors**

Please indicate the extent of your agreement with the following statements according to your experience in English class.

Strongly Disagree/Disagree/Not sure/Agree/Strongly Agree

| **Item Chinese** | **English** |
| --- | --- |
| ***Gratitude*** | |
| 1 英语学习中有太多值得我感激的。 | I have so much to be thankful for in my English learning. |
| 2 如果让我把所有心怀感激的事物都列出来，那会是一张很长的清单。 | If I had to list everything that I felt grateful for, it would be a very long list. |
| 3 当我回顾英语学习经历，几乎没有值得我感激的。 | When I look back on my English learning journey, I don’t see much to be grateful for. |
| 4 我感激在英语学习中支持过我的人。 | I am grateful to those who have supported me in my English learning. |
| 5 随着时间推移，我更加感激影响我英语学习的人和事。 | As time goes by, I appreciate more those who have influenced my English learning. |
| 6 我需要很长时间才能想出英语学习中要感激的人或事。 | It takes me a long time to feel grateful for something or someone in my English learning. |
| ***Control appraisal*** | |
| 1 我在英语课上越努力，成绩就越好。 | The more effort I put into English courses, the better I do in them. |
| 2 我认为在英语学习中的表现主要取决于自己。 | I see myself as largely responsible for my performance throughout English learning. |
| 3 我对自己在英语课上的表现有很强的控制力。 | I have a great deal of control over my academic performance in English class. |
| ***Value appraisal（Intrinsic value and Extrinsic value）*** | |
| ***Intrinsic value*** |  |
| 1 我认为在英语课上学到的知识可以应用于其他学科中。 | I think the knowledge I learned in English class can be applied to other subjects. |
| 2 我喜欢在英语课上学到的知识。 | I like what I am learning in English class. |
| 3 我觉得在英语课上学到的东西很有趣。 | I think that what we are learning in English class is interesting. |
| ***Extrinsic value*** |  |
| 1 在英语课上取得好成绩对我来说是最令人满意的事。 | Getting a good grade in this class is the most satisfying thing for me. |
| 2 我在英语课上主要关心的是取得好成绩。 | My main concern in English class is getting a good grade. |
| 3 我想在英语课上表现好是因为向他人展示我的能力很重要。 | I want to do well in English class because it is important to show my ability to others. |
| ***Enjoyment（Enjoyment Private, Enjoyment Teacher and Enjoyment Atmosphere）*** | |
| ***Enjoyment Private*** |  |
| 1 我不厌倦英语学习。 | I never get tired of learning English. |
| 2 我享受英语学习。 | I enjoy learning English. |
| 3 学英语过程中，我学了很多有趣的事情。 | During the process of learning English, I learned many interesting things. |
| 4 在班里，我为自己的英语成绩感到自豪。 | In the class, I am proud of my English grades. |
| 5 我认为学习英语很有趣。 | I think learning English is very interesting. |
| ***Enjoyment Teacher*** |  |
| 1 英语老师总是鼓励我们。 | The English teacher always encourages us. |
| 2 英语老师很友善。 | The English teacher is very friendly. |
| 3 英语老师总是支持我们。 | The English teacher always supports us. |
| ***Enjoyment Atmosphere*** |  |
| 1 周围英语学习的氛围很好。 | The English learning atmosphere around here is very good. |
| 2 我身边有很好的英语学习氛围。 | There is a very good English learning atmosphere around me. |
| 3 我们有紧密的英语学习小组。 | We have a close-knit English study group. |
| ***Agentic engagement*** | |
| 1 在英语课上，我总是主动提问题。 | I actively ask questions in English class. |
| 2 我主动告诉英语老师我喜欢什么和不喜欢什么。 | I take the initiative to tell my English teacher what I like and dislike. |
| 3 我主动让英语老师知道我对英语哪些方面感兴趣。 | I voluntarily let my English teacher know which aspects of English interest me. |
| 4 在英语课上，我经常主动表达我的观点。 | I actively express my views in English class. |
| 5 我会就如何使英语课堂变得更好提出建议。 | I offer suggestions on how to make English classes better. |
